# A Man With Progressive Chorea and Abnormal Trunk Movements

**DOI:** 10.7759/cureus.62004

**Published:** 2024-06-09

**Authors:** Ramkumar Sugumaran, Ragavendar Bhuvaneswaran

**Affiliations:** 1 Neurology, Jawaharlal Institute of Postgraduate Medical Education and Research, Puducherry, IND

**Keywords:** rubberman, trunk extension, head drops, neuroacanthocytosis, chorea-acanthocytosis

## Abstract

Neuroacanthocytosis (NA) syndromes are a group of rare genetic disorders characterized by the presence of abnormally shaped red blood cells (acanthocytes) and the progressive degeneration of the basal ganglia, leading to various neurological and systemic symptoms. The “rubber man” gait, characterized by distinctive flexions of the neck (manifesting as head drops) and the trunk, is seen in advanced chorea-acanthocytosis. A 35-year-old male patient presented with progressive abnormal movements affecting his limbs and face, along with dysphagia resulting from involuntary protrusion of the tongue and biting of the cheeks and lips over the past three years. He used to place the food on the back of his tongue and throw his head back to begin swallowing. He also kept a towel in his mouth to absorb saliva and prevent cheek and lip biting. The neurologic examination revealed generalized chorea, severe orolingual dystonia (eating dystonia), and sudden loss of tone while walking, resulting in flexion of the trunk followed by extension. We believe that these features could serve as definitive clinical indicators for chorea-acanthocytosis, providing valuable diagnostic insights, especially when accompanied by self-mutilatory mouth movements or feeding-related tongue dystonia.

## Introduction

Neuroacanthocytosis (NA) syndromes represent a group of genetically defined disorders characterized by the presence of acanthocytosis in red blood cells and the gradual degeneration of the basal ganglia [1]. Yamamoto et al. first used the term neuroacanthocytosis in 1982 to refer to the co-occurrence of acanthocytes with neurological diseases [2]. These disorders are exceptionally rare, and there is a significant likelihood of underdiagnosis, with an estimated prevalence ranging from less than one to five cases per 1,000,000 individuals for each specific disorder [1]. The two primary NA syndromes are X-linked McLeod syndrome and autosomal recessive chorea-acanthocytosis (ChAc). They exhibit diverse clinical presentations, encompassing movement disorders, neuropathy, seizures, autonomic features, dementia, and psychiatric symptoms [3]. As the disease advances, a distinct phenotype typically emerges in most patients, featuring chorea, a distinctive "feeding dystonia" characterized by tongue protrusion, unintentional lip- and tongue-biting, involuntary vocalizations, orofacial dyskinesias, and dysarthria. Individuals with ChAc may display a characteristic "rubber man" gait, characterized by truncal instability and abrupt truncal movements [1].

The term "rubber man" gait was introduced by Schneider in 2010 based on observations of four patients [4]. The authors noted that the abrupt and dramatic trunk and head movements occasionally resembled a sudden loss of tone similar to negative myoclonus. In more extreme situations, these motions may result in drops from the waist down, which would bring the head down onto the lap. Characteristic head drops with ballistic bending of the neck were seen in milder cases. We hereby demonstrate our observations with a video of characteristic flexion and extension of the neck and trunk with orolingual dystonia in a person with advanced NA. These features could serve as distinctive clinical indicators of ChAc, potentially offering valuable insights for diagnosis, especially when accompanied by additional symptoms such as self-mutilation involving mouth movements or abnormal tongue control during feeding.

## Case presentation

A 35-year-old man, born from a non-consanguinous marriage with no significant family medical history, presented with gradually progressive abnormal limb and facial movements, dysphagia due to involuntary tongue protrusion, and cheek and lip biting for the past three years. He used to place the food on the back of his tongue and throw his head back to begin swallowing. He also kept a towel in his mouth to absorb saliva and prevent cheek and lip biting. The neurologic examination revealed generalized chorea, severe orolingual dystonia (eating dystonia), and an abrupt reduction in tone when walking, resulting in trunk flexion followed by extension (Video 1). This phenomenology is called the “rubber man” gait. The sudden loss of tone while walking, resulting in trunk flexion followed by extension, resembles a “rubber man” [4]. He made noticeable backward trunk movements, sometimes causing his head to bump against the wall behind him.

**Video 1:** Rubber man gait The video shows generalized chorea, severe orolingual dystonia (eating dystonia), and an abrupt reduction in tone when walking, resulting in trunk flexion followed by extension.

The results of the hemogram, serum electrolyte, renal, and liver function tests were within normal limits (Table 1).

**Table 1:** Investigations Investigations were within normal limits except for the peripheral smear, which showed the presence of acanthocytes. WBC: white blood cell; NCCT: non-contrast computed tomography.

Lab Parameter	Values
Haemoglobin	13.5 grams per deciliter (g/dL)
Total WBC count	7,800 cells per cubic millimeter (cmm)
Platelets	300,000 per cmm
Peripheral smear	Normocytic normochromic red blood cells with acanthocytes
Urea	18 mg/dL
Creatinine	0.54 mg/dL
Sodium	136 mEq/L
Potassium	4.43 mEq/L
Calcium	9.13 mEq/L
Magnesium	1.91 mEq/L
Ceruloplasmin	21.1 mg/dL
Serum albumin	4.2 g/dL
Aspartate aminotransferase	21 U/L
Alanine aminotransferase	22 U/L
Creatine phosphokinase (CPK)	95 U/L
NCCT brain	Normal
ECG	Normal
Chest X-ray	Normal
Echocardiogram	Normal
Urine routine	Within normal limits

**Figure 1:**
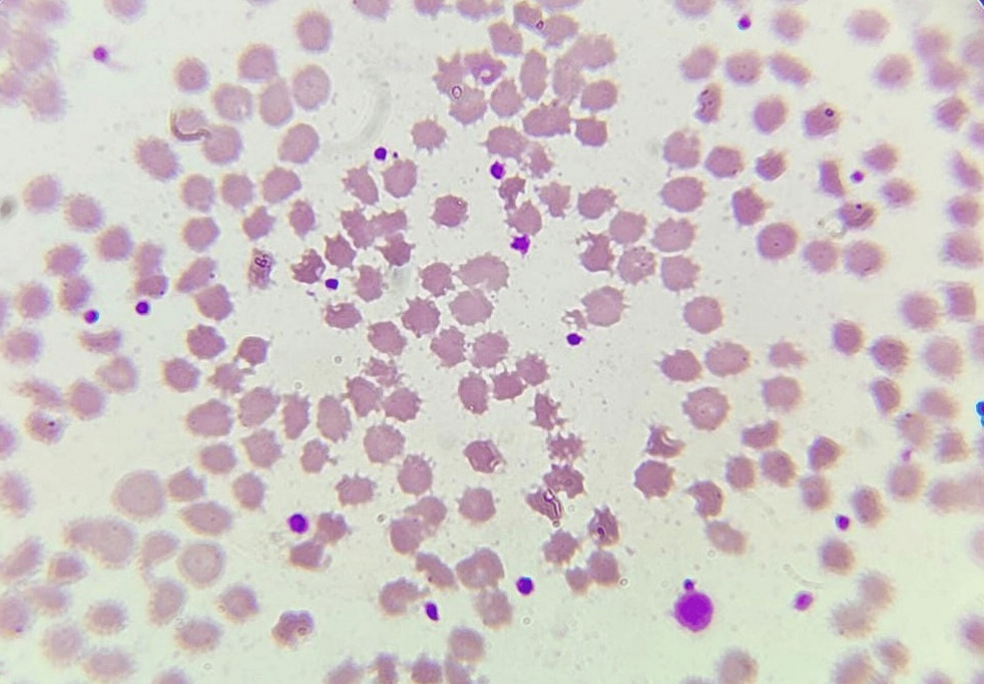
Peripheral smear showing the presence of acanthocytes The peripheral smear shows irregular or unevenly arranged spicules (acanthocytes).

**Figure 2:**
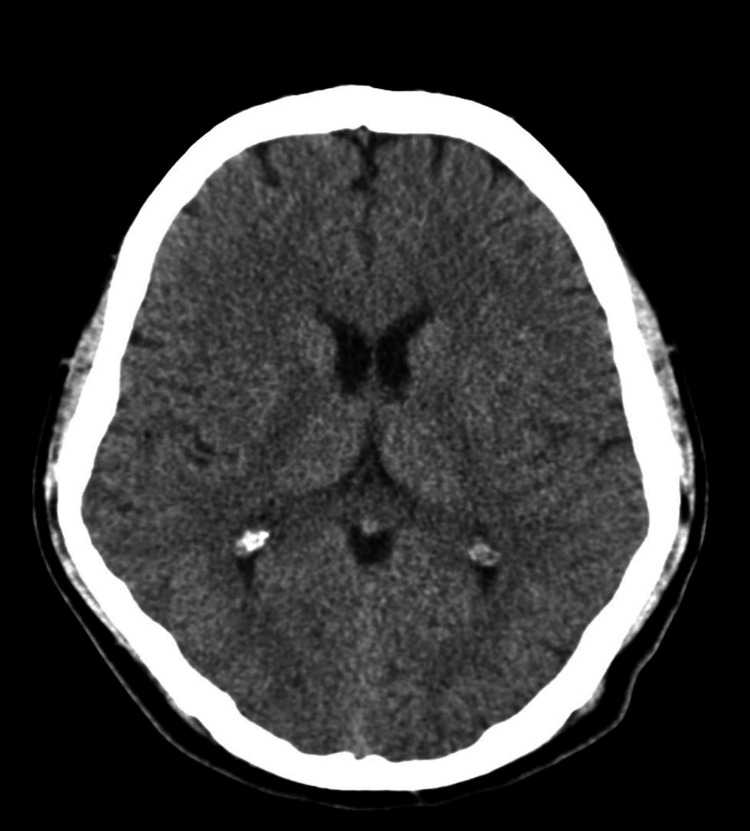
NCCT brain The NCCT brain was normal, with a preserved caudate nucleus. NCCT: non-contrast computed tomography.

The peripheral smear indicated that 10% of red blood cells (RBCs) in our patient were acanthocytes (Figure 1). The computerized tomography (CT) scan of the brain was normal (Figure 2), and genetic testing was not done due to financial constraints. The patient was treated with tetrabenazine, and there was moderate improvement in dystonic symptoms, but he still required one-person support for his activities of daily living. Subsequently, the patient was lost to follow-up.

## Discussion

NA syndromes present with a wide range of clinical features, including movement disorders, neuropathy, seizures, autonomic dysfunction, dementia, and psychiatric symptoms. Individuals with ChAc may exhibit a distinctive "rubber man" gait, characterized by trunk instability and sudden, jerky trunk movements. The term "rubber man" gait was coined by Schneider in 2010 based on observations of four patients. This gait involves a sudden loss of muscle tone while walking, leading to trunk flexion followed by extension, resembling a "rubber man" [4]. Similar myoclonus-like movements can also manifest in myoclonic illnesses such as Unverricht-Lundborg disease, where myoclonus is more widespread but the age of onset is in early adolescence between 7 and 14 years of age [5], and the overall phenomenology is much more complex in NA [6].

In ChAc, a comparable gait termed "stutter-step" has been identified. This gait is distinguished by characteristics such as a shortened length and tempo of steps, hesitancy (particularly in the heel-off terminal stance phase), increased knee hyperflexion in the mid-stance phase, and unequal foot weight distribution in the loading or foot-flat phase [7]. The majority of ChAc patients undergo the progression of generalized chorea, with a minority potentially developing Parkinsonism. Seizures, typically of a generalized nature, serve as the initial presentation of the disease in at least one-third of patients. Memory loss and executive function impairment are common occurrences, though not universal. Alongside orofaciolingual dystonia, limb dystonia is a prevalent feature. Psychiatric symptoms are also common and can include obsessive-compulsive disorder or psychosis resembling schizophrenia [1]. However, it is important to note that acanthocytosis can show variability, and acanthocytes on a peripheral blood smear are not always necessary for the diagnosis of these illnesses [1].

The combination of diluted blood with wet blood preparation demonstrated high specificity (0.98), successfully identifying all genetically confirmed ChAc patients. This method is cost-effective, easily accessible, and provides high specificity and sensitivity for detecting clinically significant acanthocytosis. It is recommended to use isotonically diluted blood samples with an unfixed wet blood preparation to effectively identify significant acanthocytosis in movement disorders [8]. Protein assays or genetic testing stands out as a more sensitive laboratory parameter in detecting ChAc [4]. Confirming the diagnosis of ChAc often involves investigating various mutations in the *VPS13A* gene, a 73-exon gene on chromosome 9 that codes for the protein chorein [9]. Because of the large size of the *VPS13A* gene and the variety of places where mutation sites might be found, confirmatory DNA analysis can be difficult [9]. Chorein, coded by *VPS13A*, plays a role in the sorting of intracellular proteins, yet its specific physiological roles are unknown.

Preliminary studies of post-mortem human brain tissue suggest lipid metabolism dysfunction at the subcellular level. The proteins VPS13A and XK are involved in bulk lipid transfer at membrane contact sites, where they closely interact, forming a protein complex at the junctions between the endoplasmic reticulum and the cellular membrane system. VPS13A, along with other VPS13 proteins (B-D), belongs to the newly recognized superfamily of bridge-like lipid transfer proteins (BLTPs). Mutations in other *VPS13* genes cause neurodegenerative (*VPS13C*, *VPS13D*) or neurodevelopmental (*VPS13B*) disorders. While the exact pathophysiology remains unknown, it is possible that VPS13A and XK diseases, along with related conditions, represent a new group of disorders with a common mechanism involving impaired bulk lipid transport [10].

A Western blot can be used to show that there is no chorein present in the erythrocytes. In our case, testing for chorein was not conducted due to financial constraints. In NA syndromes, the neurodegenerative process primarily impacts the putamen, caudate nucleus, and globus pallidus. In ChAc, the degeneration extends to involve the thalamus and substantia nigra as well [1]. The differential diagnosis of chorea in early adulthood includes Huntington’s disease, spinocerebellar ataxia, HDL2 (Huntington’s disease-like), neuroferritinopathy, Wilson’s disease, NA syndromes, VPS13A‑ and XK‑disease/McLeod, aceruloplasminemia, Niemann‑Pick type C disease, drug-induced, autoimmune (anti-NMDAR encephalitis) causes, and Lesch-Nyhan syndrome [11]. A distinctive feature of ChAc is the existence of self-mutilating habits, such as biting one's lips or tongue, or other self-mutilating actions, such as finger biting. The occurrence of such behaviors, along with the presence of acanthocytes in the peripheral smear, strongly suggests ChAc. It is worth noting that while boys with Lesch-Nyhan syndrome may also exhibit self-mutilation, the onset of symptoms in these cases typically occurs at a much younger age.

Treatment for NA is mainly symptomatic and supportive, as there is currently no cure. The management strategies focus on alleviating symptoms and improving the quality of life. Medications such as antipsychotics, tetrabenazine, or deutetrabenazine may be used to manage chorea and other involuntary movements. Botulinum toxin injections can be effective for focal dystonia or other specific movement issues. Antidepressants, antipsychotics, and mood stabilizers can help manage psychiatric symptoms such as depression, anxiety, and psychosis. Speech therapy can assist with communication difficulties. Swallowing difficulties may require dietary modifications, thickened liquids, or even feeding tubes in severe cases. Ensuring adequate nutrition is crucial, especially if swallowing is compromised. A dietitian can provide guidance on appropriate nutritional strategies. Physical and occupational therapy can help maintain mobility and independence, improve muscle strength, and manage spasticity. Management of orthostatic hypotension and other autonomic symptoms may involve medications, lifestyle changes, and dietary adjustments.

For patients and families, genetic counseling can provide information on the hereditary nature of the disorder and discuss potential risks for future generations. Given the complexity of NA, a multidisciplinary team, including neurologists, psychiatrists, speech therapists, dietitians, and physical therapists, is often necessary for comprehensive care [12]. While these treatments can alleviate some of the symptoms and improve quality of life, ongoing research is essential to develop more effective treatments for NA syndromes.

## Conclusions

In cases of ChAc, distinctive signs such as characteristic head drops, axial extension, rubber man gait, and feeding dystonia are noteworthy. Recognizing these specific features can be crucial in differentiating ChAc from other disorders within the NA syndrome.
